# An experimental method for efficiently evaluating the size-resolved sampling efficiency of liquid-absorption aerosol samplers

**DOI:** 10.1038/s41598-022-08718-8

**Published:** 2022-03-18

**Authors:** Jianshu Guo, Xinying Zheng, Tongtong Qin, Meng Lv, Wei Zhang, Xiaolin Song, Hongying Qiu, Lingfei Hu, Lili Zhang, Dongsheng Zhou, Yansong Sun, Wenhui Yang

**Affiliations:** 1 State Key Laboratory of Pathogen and Biosecurity, Beijing Institute of Microbiology and Epidemiology, Beijing, 100071 China; 2 Laboratory Animal Center, Academy of Military Medical Science, Beijing, China

**Keywords:** Environmental sciences, Natural hazards

## Abstract

Aerosol samplers are critical tools for studying indoor and outdoor aerosols. Development and evaluation of samplers is often labor-intensive and time-consuming due to the need to use monodisperse aerosols spanning a range of sizes. This study develops a rapid experimental methodology using polydisperse solid aerosols to evaluate size-resolved aerosol-to-aerosol (AtoA) and aerosol-to-hydrosol (AtoH) sampling efficiencies. Arizona Test Dust (diameter 0.5–20 µm) was generated and dispersed into an aerosol test chamber and two candidate samplers were tested. For the AtoA test, aerosols upstream and downstream of a sampler were measured using an online aerodynamic particle sizer. For the AtoH test, aerosols collected in sampling medium were mixed with a reference sample and then measured by the laser diffraction method. The experimental methodology were validated as an impressive time-saving procedure, with reasonable spatial uniformity and time stability of aerosols in the test chamber and an acceptable accuracy of absolute mass quantification of collected particles. Evaluation results showed that the AGI-30 and the BioSampler sampler had similar size-resolved sampling efficiencies and that efficiencies decreased with decreasing sampling flow rate. The combined evaluation of AtoA and AtoH efficiency provided more comprehensive performance indicators than either test alone. The experimental methodology presented here can facilitate the design and choice of aerosol sampler.

## Introduction

Aerosols are solid, liquid or mixed particles suspended in air that range in size from 1 nm to 100 µm which are mainly produced by natural and human activities such as soil particle suspension, plant pollen dispersal, seawater evaporation, fossil fuel combustion, industrial and agricultural production, building construction, transportation processes, even human breathing^1,2^. Those aerosols containing biological components such as bacterial cells and spores, viruses, pollen, fungi, algae, detritus, allergens and cell fragment are often referred to as biological aerosols or bioaerosols. Substances carried by aerosols can affect not only the atmosphere environment, but can also have direct adverse effects on human health^3–5^. Many studies have proved that occupational and daily environmental aerosol exposures increase the risk of acute and chronic respiratory diseases, such as asthma, allergic rhinitis, pneumoconiosis, COPD, lung cancer^6–10^. Bioaerosol particles are usually a small fraction of all aerosol particles in our surroundings, but their impact can be critical. Airborne pathogenic microorganisms contained in bioaerosol particles are highly infectious among individuals, especially in outbreaks such as the coronavirus disease 2019 (COVID-19), middle east respiratory syndrome (MERS), influenza A (H1N1), and severe acute respiratory syndrome (SARS)^11–13^. Aerosols can be carried into the middle-to-upper troposphere by appropriate conditions such as major tropical hurricanes, tropical cyclones, windblown plume and be transported over long distances^14,15^.

With the increasing desire to understand the properties of aerosols, indoor and outdoor aerosols are frequently collected for purposes such as public health, climate and other studies^16,17^. Characterization of aerosols in a given environment requires representative aerosol samples. However, particles can be lost during some physical aerosol sampling steps such as aspiration, transport, deposition and exhaust, and this will affect the representativeness of the collected sample^18,19^. Therefore, an adequate understanding about the performance of aerosol samplers is important for choosing a sampler and when characterizing aerosol samples. In recent years, increasing utilization of monodisperse and polydisperse aerosols to evaluate aerosol to aerosol (AtoA) sampling efficiency of aerosol samplers has emerged^20–24^. In those studies, environmental or artificially generated aerosols were delivered into candidate samplers. Then the particle size distribution upstream and downstream of the samplers were measured using online aerosol detection systems, such as the optical particle counter (OPC), aerodynamic particle sizer (APS) or scanning mobility particle sizers (SMPS). When using this method, reference sampler is not required as well as additional sample extraction and sample analysis. Although there are advantages to evaluating the AtoA sampling efficiency using polydisperse aerosols, this evaluation method takes no account of the effect ‘wall loss’, whereby some particles were collected on the sampler wall rather than in the sampling medium^25^. However, post-sampling analysis typically assumes the particles in the sampling medium are the total particles collected by the aerosol sampler^26–28^. In this case, evaluation of aerosol to hydrosol (AtoH) sampling efficiency may better represent the physical sampling efficiency.

A monodisperse test aerosol is conventionally used to evaluate aerosol sampler performance of AtoH sampling efficiency. In these tests, an adequate number of different-sized monodisperse aerosols, such as spherical standard polystyrene-latex (PSL) particle or liquid oleic acid particle are generated and tested in sequence, with the objective of obtaining a wide size range sampling efficiency^29,30^. Although this method results in an accurate sampling efficiency of certain sized particles, it is extremely labor-intensive and time-consuming.

In contrast, a size-resolved AthH sampling efficiency evaluation method using polydisperse aerosols could be time-saving and low cost as compared to method using monodisperse aerosols, since the sampling efficiency of dozens of particle sizes can be determined in a single measurement. However, this method also has disadvantages, which requires expertise and additional analysis steps. Agglomerate and aggregate of aerosol particles during performance test could affects the accuracy of the final evaluation results. Only a few studies have established the methodology using polydisperse aerosols to evaluate size-resolved AtoH sampling efficiency of aerosol sampler^31,32^. In those study, the coulter counter, which is capable of measuring solid polydisperse particles, has been successfully used in size-resolved AtoH evaluation by the U.S. Environmental Protection Agency (EPA)^31^. However, the inherent feature of the coulter counter means that the measurement range of particle diameter analysis is limited by the pore size of the aperture. In that case, a laser particle analyzer is recommended because it can theoretically determine particle size characteristics in the size range of 0.01–3500 µm during a single measurement. But the laser particle analyzer is unable to directly measure the absolute concentration of test particles, which restricts its application in size-resolved AtoH evaluation.

In this work, a method is developed to use the laser particle analyzer to obtain absolute concentrations by introducing a reference sample in the detection process. Furthermore, performance testing using this laser diffraction method is done to evaluate the AtoH sampling efficiency of the AGI-30 and the BioSampler and these results were compared with the AtoA sampling efficiency performance. The results from this work highlight the potential of the laser diffraction method to conduct AtoH sampling efficiency evaluation using polydisperse aerosols. And the test results show consistency with the AtoA and AtoH test using monodisperse aerosols and other experimental studies.

## Materials and methods

### Test aerosol sampler

Two of the most often used samplers were evaluated in the present study, the AGI-30 (ACE Glass Inc.) and the BioSampler (SKC Inc.); both use a liquid collection medium to capture droplets and aerosols. They both consist of an inlet, a collection vessel, and an outlet. The inlet of the AGI-30 contains one acceleration nozzle with an inner diameter of 1.27 mm through which air is drawn at a flow rate of 12.3–12.6 l per min (LPM). The jet created by the nozzle pushes aerosol particles to the bottom of the glass vessel. The BioSampler inlet contains three tangential nozzles with inner diameters of 0.63 mm that create a swirling motion in the liquid collection medium with a sampling flow rate of 12.5 LPM. The swirling motion minimizes the chances of particle re-nebulization^33^. Typically, the initial volume of collection liquid is 20 ml for both the AGI-30 and the BioSampler.

A polycarbonate filter with a pore size of 0.4 µm (37 mm diameter, GVS Inc.) was used as the reference sampler; it consisted of a smooth, translucent surface with straight-through capillary holes across the membrane structure^34^. The holes with narrow range sizes on the uniform structure enable the filter to efficiently collect particles with diameters > 0.4 μm.

### Polydisperse test particle

The physical aerosol sampling efficiency of the candidate sampler was measured using Arizona Test Dust (ATD, Powder Technologies Inc.). A widely used and inexpensive solid aerosol particle, ATD is insoluble and easily dispersible, meeting the size-resolved evaluation requirements perfectly. For the evaluation experiment, ATD with nominal diameters of 0–3 µm, 0–10 µm and 10–20 µm were blended in a mass ratio of 1:1:2, baked at 200 ºC for 24 h and cooled to room temperature before aerosolization. Morphology of the test ATD particles before and after aerosolization were observed using scanning electron microscopy (S-3400 N, Hitachi) at an accelerating voltage of 15 kV. The aerodynamic particle size of ATD was calculated using Eq. (1). The density of ATD dust is 2.5462 g/cm^3^, and the dynamic shape factor is 1.4, as described^31^.

1 $$\begin{array}{*{20}c} {Dp_{a} = Dp_{e} \left( {\frac{{\rho_{p} }}{{\rho_{o} \chi }}} \right)^{\frac{1}{2}} ,} \\ \end{array}$$

where $Dp_{a}$ = aerodynamic particle size, µm; $Dp_{e}$ = equivalent particle size, µm; $\rho_{p}$ = particle density, g/cm^3^; $\rho_{o}$ = aerodynamic particle density, 1.0 g/cm^3^; $\chi$ = dynamic shape factor, unitless.

### Aerosol generation

Aerosolization of blended ATD was achieved using an improved rotating brush type solid aerosol generator (Beijing Huironghe Technology Co., Ltd.). The aerosol generator system was designed to disperse non-cohesive powders with particle size < 100 μm^35^. ATD was placed in the powder reservoir, uniformly compressed by a tamper, and then transported to a rotating brush at a 0.1–8 mm/min feed rate by the transportation piston. The rotating brush was used to stably deliver ATD to the dispersion nozzle and prevent the influence of highspeed airflow on ATD in the powder reservoir. Downstream of the rotating brush, a stainless-steel dispersion nozzle was used to effectively aerosolise the dispensed bulk material into discrete particles at flow rate of 20 LPM.

**Figure 1:**
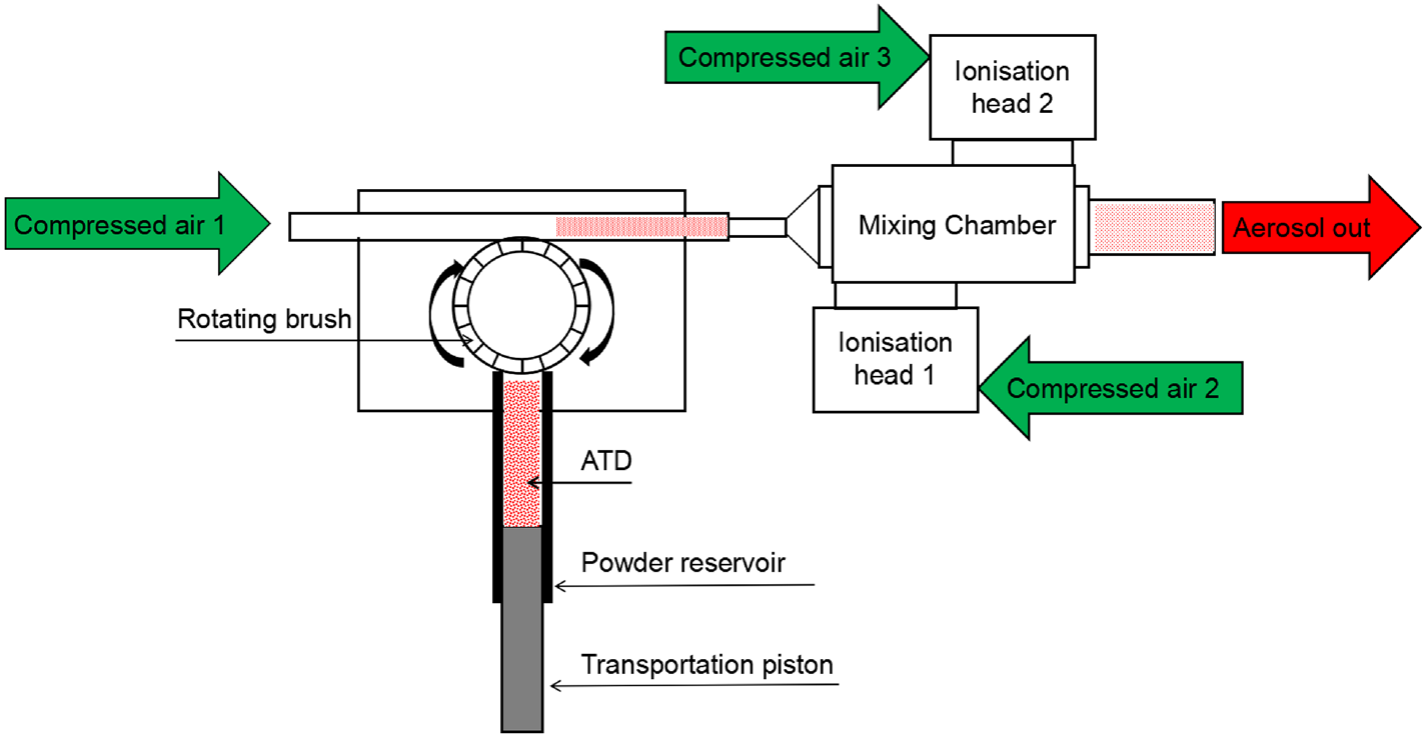
Schematic of system used to generate charge neutralized ATD aerosol. Schematic not drawn to scale.

**Figure 2:**
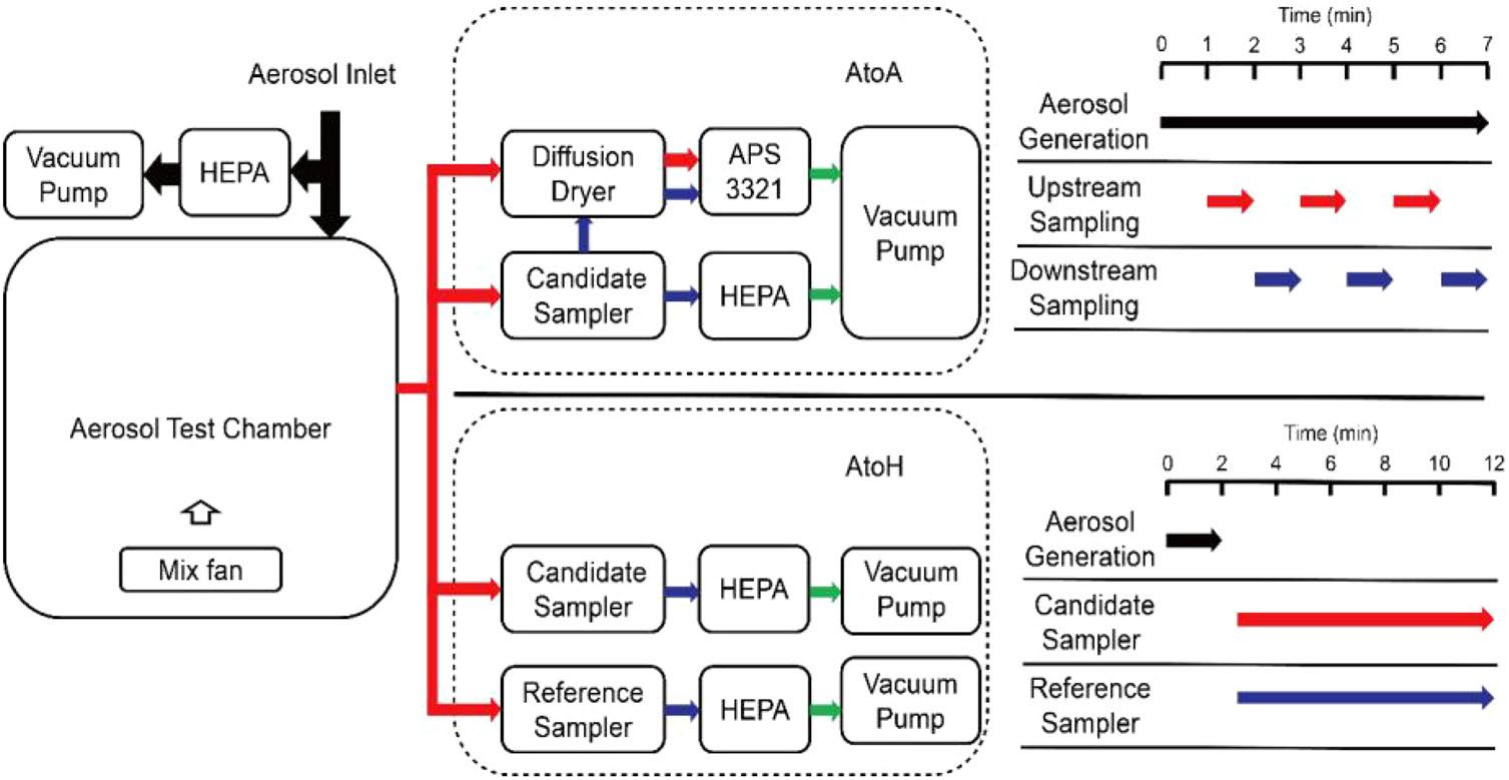
Schematic diagram of the experimental setup for measuring the AtoA and AtoH sampling efficiency of test sampler.

To remove electrical charge from aerosols and avoid particle aggregation in the air, the discrete ATD particles were sequentially passed through an electrostatic neutralizer consisting of a positive and negative ionization head and a mixing chamber (EAN-581, TOPAS Inc.). ATD aerosols particles mixed with ionized air produced by the positive and negative ionization heads and electrostatic neutralization can be achieved in the mixing chamber (Fig. 1). Then the test aerosol was divided into two parts, one part passed through the high-efficiency particulate air (HEPA) filter and the other part entered the aerosol test chamber (Fig. 2).

### Experimental setup of aerosol sampling

Aerosol sampler performance tests were conducted in a 300 L stainless aerosol test chamber (Beijing Huironghe Technology Co., Ltd.) equipped with a HEPA filtration system to provide a low background aerosol concentration and prevent contamination of the surrounding environment. The chamber also contained an aerosol injection port, several aerosol sampling ports, a 120 mm fan for aerosol mixing, and several meteorology sensors for temperature, humidity and pressure monitoring.

Evaluation of AtoA and AtoH sampling efficiency were performed using two independent experiments (Fig. 2). To evaluate AtoA sampling efficiency, the ATD aerosols were generated and mixed in the test chamber throughout the entire test process with a particle concentration of approximately 400 particles /cm^3^. A candidate sampler (AGI-30 or BioSampler) was connected to a sampling port of the test chamber, and the number-based size distribution and count concentration of ATD aerosols upstream and downstream from the candidate sampler was measured with an aerodynamic particle sizer (APS, model 3321, TSI Inc.). A diffusion dryer (model 3062, TSI Inc.) was used to remove moisture generated by the aerosol sampler. To minimize the effect of aerosol concentration changes on the results, alternate upstream and downstream sampling was conducted three times for 1 min.

To evaluation the AtoH sampling efficiency, aerosols were released into the test chamber at the maximum rate for 2 min before sampling and mixed throughout the entire tests process. Aerosols were then sampled simultaneously by a candidate sampler (AGI-30 or BioSampler) and a reference sampler (polycarbonate filter) at the same sampling flow rate for 10 min. 10 minuites was chosen in our study for a reason. After 10 min of sampling, sufficient particle concentrations can be obtained for subsequent particle analysis. Most of the aerosol particles in the aerosol test chamber can be collected and more sampling time does not contribute to higher particle concentrations in sampling media. Impact from liquid evaporation and re-aerosolization can also be negligible at sampling times of 10 minutes^36^. And the error of the manually operated sampling time is small with 10 min sampling. Collected particles of both samplers then underwent subsequent particle extraction and laser diffraction analysis.

In both the AtoA and AtoH tests, The AGI-30 and the BioSampler were evaluated separately at the sampling flow rate of 6, 9 and 12.5 LPM. Deionized water (20 ml) was used as the sampling medium for both samplers. Vacuum pumps were used respectively to control the concentration of ATD aerosols in the test chamber and the sampling flow rate of the tested samplers in both tests. In addition, The AtoA sampling efficiency of polycarbonate filter was also evaluated at the same sampling flow rates. Each experiment was repeated three times.

### Validation of sampling methods

Evaluation of AtoA sampling efficiency requires time stability of aerosol concentration between the upstream and downstream sampling points of the candidate sampler over different sampling periods. To assess the time stability of aerosol concentration in the AtoA test, a short straight tube with inner diameter of 6.5 mm was used to replace the candidate sampler at a sampling flow rate of 12.5 LPM. Aerosols upstream and downstream from the tube were measured with APS as described above.

Different from the AtoA test, evaluation of AtoH sampling efficiency requires spatial uniformity of aerosol concentration in the test chamber. Therefore two polycarbonate filter samplers were placed in opposite direction, and aerosols were sampled synchronously by the two samplers at a flow rate of 12.5 LPM for 10 min. Collected particles of both samplers were then extracted and analyzed as detailed below. All of the validation experiments were repeated three times.

### Particle extraction

To completely extract the collected ATD particles from the polycarbonate filter membrane, the following extraction steps were performed: the filter membrane was placed into 15 ml of prefiltered 1% Tween20 (Sigma-Aldrich), vigorously shaken for 10 s and then vortexed for 1 min using a commercially available vortex mixer. Three cycles of these extraction steps were performed and the eluents were mixed for subsequent particle analysis. Recovery efficiency of the particle extraction from polycarbonate filter were (99.78 ± 0.13) % as determined by weighing (Table S1). For AGI-30 and BioSampler, the sampling liquids were collected and the collection vessels were rinsed twice with 15 ml deionized H_2_O. The sampling liquid and rinsing fluid were mixed together. Particles remaining on the inner wall of the sampler are also extracted and suspended in 40 ml deionized H_2_O. Tween20 was added to the mixture with an operating concentration of 1% (w/w) to disperse ATD particles before particle analysis.

### Laser diffraction method for particle analysis

A laser diffraction method was set up to analyze ATD particle suspensions collected in the AtoH tests. Mass-based particle size distribution of the test sample was measured with a laser particle analyzer (HELOS + CUVETTE, Sympatec Inc.) based on the laser diffraction principles^37^. To estimate the absolute mass concentration of the test sample, additional measurements were performed by following the steps below: A suspension containing 0.5 mg/ml ATD particles (10–20 μm) and 1% Tween20 was prepared as a reference sample. Three replicate size distribution measurements of a test sample and a reference sample were done respectively, followed by mixing of the test sample with 1 ml of the reference sample and then another three replicate size distribution measurements of the mixed sample were conducted.

Theoretically, the particle size distribution of the mixed sample can be fit using the particle size distribution and mass concentration data of the test sample and reference sample. Equation (2) was used to calculate the variation between the fitted and the measured particle size distribution of the mixed sample. Calculation of the variation value (Eq. 2, below) under various hypothetical test ATD concentrations was conducted using the statistical software R. The hypothetical value of the test ATD mass concentrations that minimized the variation value was taken as the fitted result of the test ATD suspension.

2 $$\begin{array}{*{20}c} {Var = \sum \left( {\frac{{p_{sample,i} \times c_{sample} \times v_{sample} + p_{reference,i} \times c_{reference} \times v_{reference} }}{{c_{sample} \times v_{sample} + c_{reference} \times v_{reference} }} - p_{mix,i} } \right)^{2} , } \\ \end{array}$$

where $Var$ = variation between the fitted and the measured particle size distribution, unitless; $p_{sample,i}$ = percentage of collected particles with particle size of i μm, (%); $c_{sample}$ = concentration of collected particle suspension, (mg/ml); $v_{sample}$ = volume of collected particles into the sample cup, ml; $p_{reference,i}$ = percentage of reference particles with particle size of i μm, (%); $c_{reference}$ = concentration of reference particle suspension, (mg/ml); $v_{reference}$ = volume of reference particles into the sample cup, ml; $p_{mix,i}$ = percentage of mixed particles with a particle size of i μm, (%).

### Validation of laser diffraction method

ATD suspensions (0–10 μm) with total particle masses of 0.25, 0.5, 1 and 2 mg were used to estimate the accuracy of the fitting method of mass quantification. The particle size distributions of the test, reference and mixed ATD suspensions were measured by the laser particle analyzer, and fitted mass of test ATD suspensions were calculated by R. All four groups of experiments were repeated three times.

### Sampling efficiency calculation

Following the distribution results of downstream and upstream measures by APS, the candidate sampler’s AtoA sampling efficiency, $\eta_{AtoA} ,$ of each discrete particle size was calculated as follows:

3 $$\begin{array}{*{20}c} {\eta_{AtoA,i} = 1 - \frac{{N_{down,i} }}{{N_{up,i} }},} \\ \end{array}$$

where $\eta_{AtoA,i}$ = AtoA sampling efficiency of the test sampler with particle size of i μm, when sampling ATD aerosol; $N_{down,i}$ = particle counts downstream of the test sampler with particle size of i μm, when sampling ATD aerosol; $N_{up,i}$. = particle counts upstream of the test sampler with particle size of i μm, when sampling ATD aerosol.

The candidate sampler’s AtoH sampling efficiency of each discrete particle size was calculated based on the laser diffraction analysis results by the following equation:

4 $$\begin{array}{*{20}c} {\eta_{AtoH,i} = \frac{{c_{candidate,i} \times v_{candidate} \times V_{reference} }}{{c_{reference,i} \times v_{reference} \times V_{candidate} }} ,} \\ \end{array}$$

where $\eta_{AtoH,i}$ = AtoH sampling efficiency of the candidate sampler with particle size of i μm, when sampling ATD aerosol; $c_{candidate,i}$ = particle concentration of the collected liquid from the candidate sampler with particle size of i μm, mg/ml; $v_{candidate}$ = volume of the collected liquid from candidate sampler with rticle size of i μm, ml; $V_{candidate}$ = sampling air volume of candidate sampler, L; $c_{reference,i}$ = particle concentration of the collected liquid from reference sampler with particle size of i μm, mg/ml; $v_{reference}$ = volume of the collected liquid from reference sampler with particle size of i μm, ml; $V_{reference}$ = sampling air volume of reference sampler, L.

### Validation of sampling efficiency of test aerosol samplers

**Figure 3:**
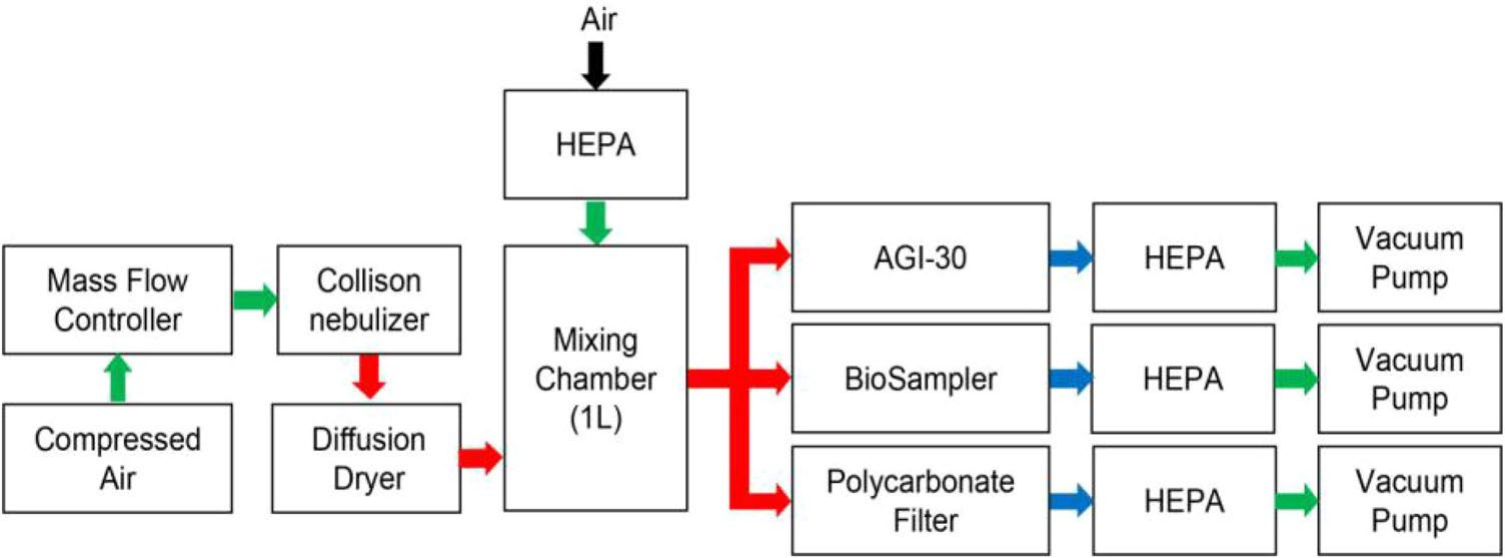
Schematic diagram of the experimental setup to evaluate AtoH sampling efficiency of AGI-30 and BioSampler by fluorescent polystyrene-latex (FPSL).

To evaluate AtoH sampling efficiency of AGI-30 and BioSampler by monodisperse aerosols, the green fluorescent polystyrene-latex particles (Fluoro-Max,Thermo) aerosols with particle size of 0.77, 1.1 and 1.9 μm were generated by collision nebulizer at flow rate of 2 LPM and dried by diffusion dryer (Fig. 3). After mixed with clean air in mixing chamber, FPSL aerosols were sampled by AGI-30, BioSampler and polycarbonate filter for 10 min. Then FPSL particles were extracted from aerosol samplers and its fluorescence intensity was measured by Qubit 4 (Thermo) with excitation wavelength at 470 nm. Sampling efficiency at sampling flow rate of 6, 9 and 12.5LPM was test. Three replicate tests were performed for each flow rate. And the recovery efficiency of extracting monodisperse particles was tested, the result indicated that particles collected on polycarbonate filter membranes were easily extracted (Table S2).

## Results

### Size distribution and particle morphology of ATD

**Figure 4:**
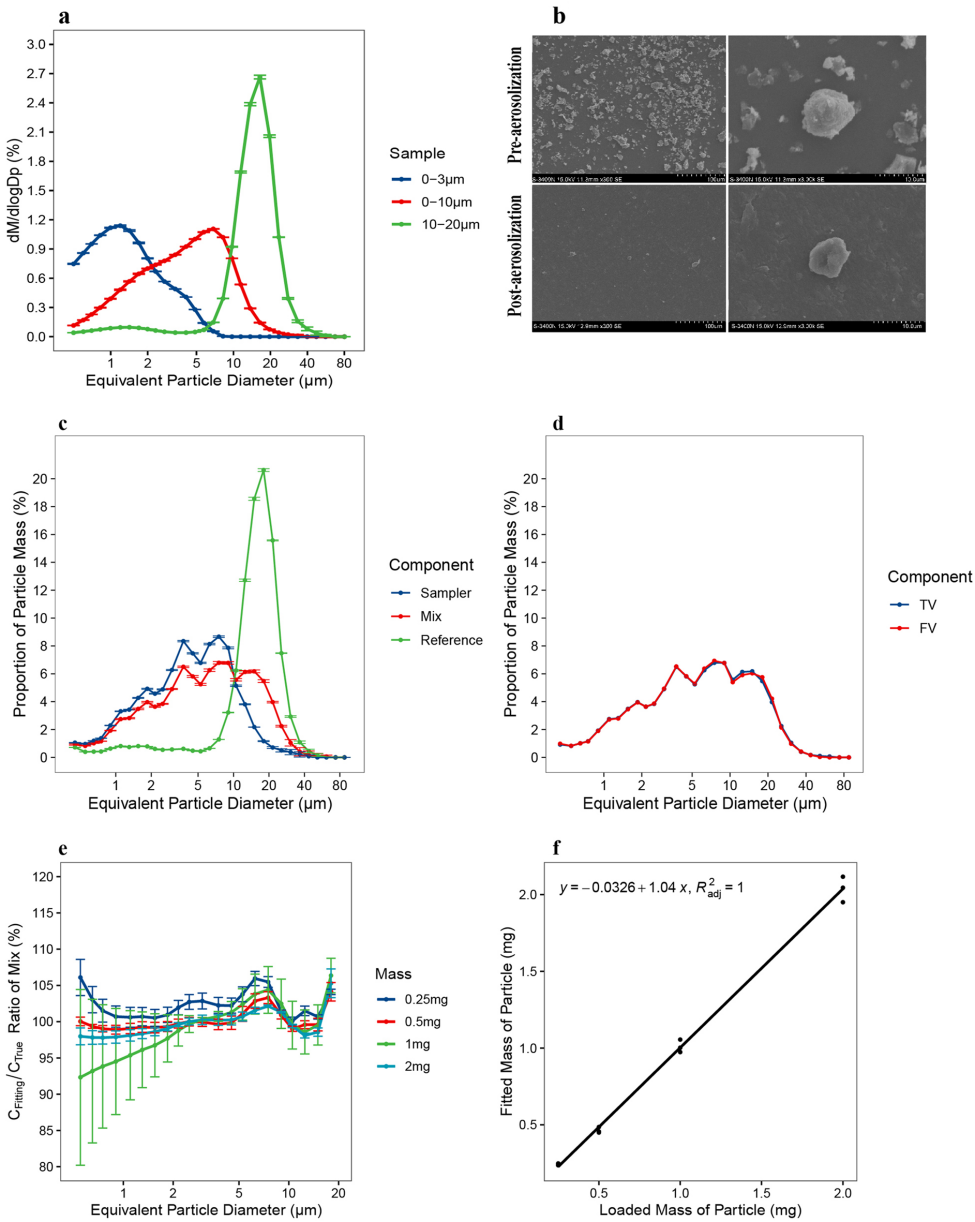
Evaluation of test particle properties and mass quantitative accuracy of ATD. (**a**) Size distribution of ATD used in this study; note logarithmic scale was used for all x-axes. (**b**) SEM photograph of blended ATD before and after aerosolization. (**c**) Typical size distribution of different component in single particle mass measurement. (**d**) Typical size distribution of true value (TV) and the fitting value (FV) of mix suspension. (**e**) Three independent experiment results of ratio between fitted value and true value of mix suspension for different loaded mass of ATD (f) Linear regression between loaded mass and fitted result of mass. Log-scale was used for the x-axis of particle size distribution plots and error bars indicate SD of three replicate measurements.

The mass median diameter (MMD) of test ATD samples were 1.1, 4.1 and 14.9 μm for 0–3 µm, 0–10 µm and 10–20 µm ATD, respectively (Fig. 4a). The scanning electron microscopy (SEM) reveals the irregular shape of ATD particles (Fig. 4b). It can be observed in SEM photo that the agglomeration and aggregation of ATD particles existed before the processes of baking, aerosolization and electrostatic neutralization. But after the above processes, the agglomeration and aggregation could no longer be observed, which indicated that the ATD particles were well dispersed in this study.

### Accuracy of laser diffraction method

To estimate the accuracy of the laser diffraction method for mass quantification, 0–10 μm ATD suspensions with total particle masses of 0.25, 0.5, 1 and 2 mg were used to conduct quantitative analyses with three replicates each. Particle size distributions of test, reference and mixed ATD suspensions were differed for one replicate experiment of 2 mg, this was key for validity of further quantitative analysis (Fig. 4c). The remaining eleven results had similar differences between size distributions of the three suspensions (data not shown). Further results show that the fitted distribution and measured distribution of mixed suspensions showed a high degree of consistency for the 2 mg ATD test samples (Fig. 4d).

For all twelve results, the fitted and the measured mixed sample distribution agreed well for the 0.45—16.9 μm equivalent particle size range, where the maximum test range was 0.45—87.5 μm (Fig. 4e). For all tested particle sizes, the mean coefficient of variation (CV) for accuracy ratios between fitted and measured estimates of mixed ATD suspensions were 4.9%, 3.9%, 4.0% and 3.5% for loading masses of 0.25, 0.5, 1 and 2 mg, respectively. Fitted results of ATD mass showed strong correlations with the actual loaded mass across ranges from approximately 0.25–2 mg, which covers the optical density range of 5–40% (the maximum measurement range is 1–50%) in the laser particle analyzer (Fig. 4f). A simple linear regression between the fitted mass and loaded mass gave a slope of 1.04 and an intercept of -0.03 mg. The average deviation from true loaded mass was -5.2%, 7.0%, 4.7% and 3.6% for loading masses at 0.25, 0.5, 1 and 2 mg, respectively. The test results show that the absolute mass quantitative ability of our method is accurate for purposes of determining the mass concentration of collected aerosols. Among replicate measurements, the CV for each fitted mass was 17.5%, 0.8%, 8.9% and 5.8% for loading mass at 0.25, 0.5, 1 and 2 mg, respectively, suggesting that an acceptable repeatability was achieved at the total loading mass range from 0.5–2 mg, which covers the optical density range of 10–40% in the laser particle analyzer. During subsequent tests, the optical concentrations of the test samples were always within this interval.

### Performance of aerosol test chamber and sampling methods

**Figure 5:**
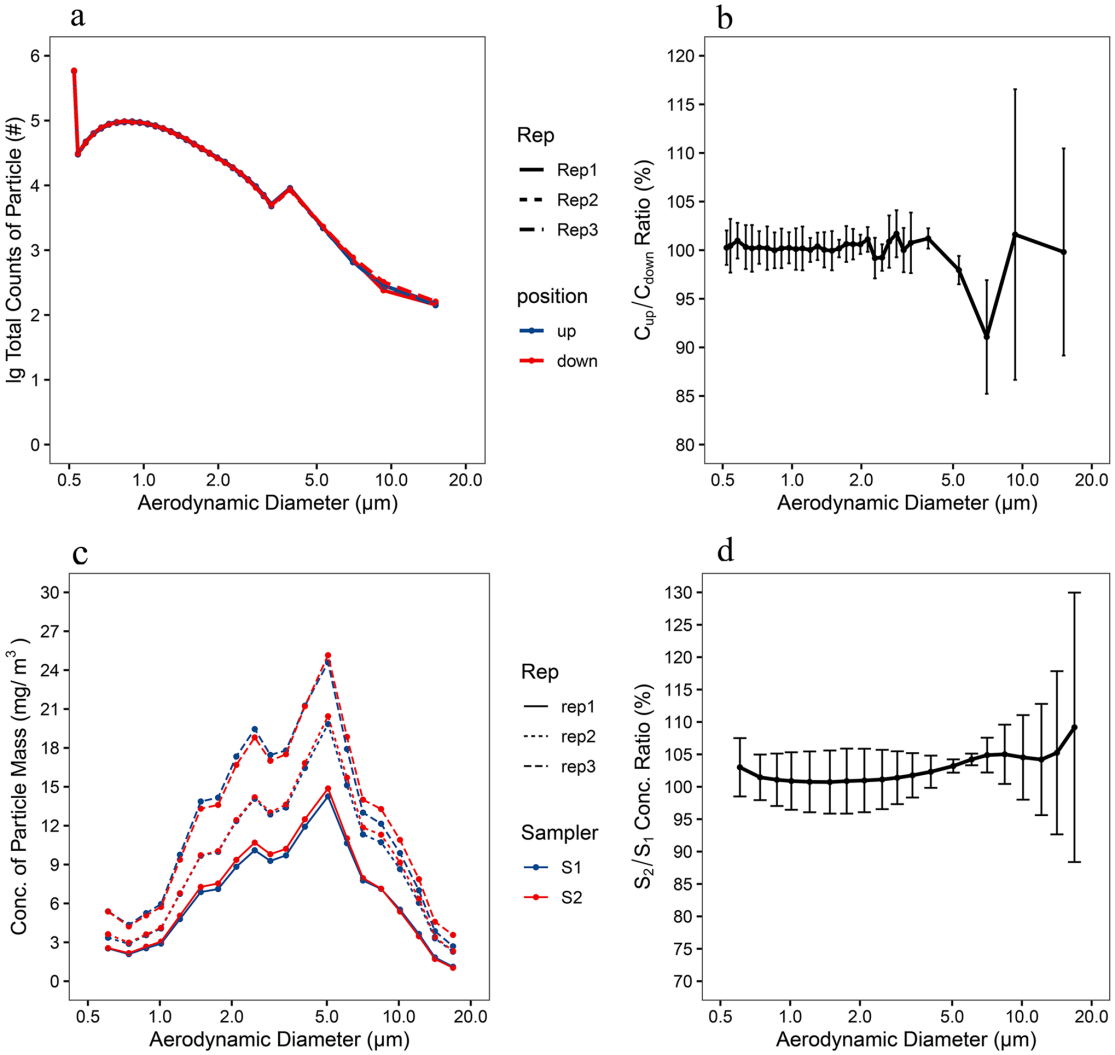
Performance of aerosol test chamber and experiment setup under evaluation of AtoA and AtoH test. (**a**) Size distribution of particles during upstream (up) and downstream (down) aerosol of test aerosol sampler. (**b**) Ratio between total counts of particle upstream and downstream of test aerosol sampler. (**c**) Size distribution of particles collected at two sampling ports of the aerosol test chamber by two filter samplers (S1, S2) at flow rate of 12.5 LPM. (**d**) Ratio between mass concentration of particle collected by two filter samplers (S1, S2) at flow rate of 12.5 LPM. Log-scale was used for the x-axis of particle size distribution plots and error bars indicate SD of three replicate measurements.

In all three replicate experiments, the total counts of particle with size range in 0.5-20 μm measured by APS were 10^5.77±0.00^ to 10^2.17±0.02^ for upstream aerosol and 10^5.76±0.01^ to 10^2.17 ±0.03^ for downstream aerosol (Fig. 5a). Across all tested particle sizes (0.5–20 μm), the mean ratio of total counts between upstream and downstream were all between 90%-110% and the CV of total count ratios between upstream and downstream was all less than 12%, with a mean value of 2.18% for all particle size ranges (Fig. 5b). These results indicate that sufficient accuracy and reproducibility of aerosol concentration during different upstream and downstream sampling periods can be obtained with this experimental evaluation setup for AtoA sampling efficiency.

Unlike the evaluation of AtoA sampling efficiency, evaluation of AtoH sampling efficiency is determined by comparing the mass-based size distribution of particles collected by the candidate sampler relative to the reference sampler. Particles collected in both filter samplers ranged in size from 0.6 to 16.9 µm were effectively measured by laser particle analyzer (Fig. 5c). The particle mass concentrations of ATD aerosol in three independent tests by two reference samplers were (189.94 ± 57.89) mg/m^3^ and (195.01 ± 57.22) mg/m^3^. The total aerosol concentration variations of each experiment were due to the the uncertainty of ATD aerosol generation, but for each experiment a reference sampler was set up and the sampling efficiency evaluated was the relative sampling efficiency to the reference sampler, thus avoiding the impact of differences in the total aerosol mass for each experiment. Across 0.6–16.9 µm tested particle sizes, the mean ratio of mass concentrations between two filter samples was 102.84% and the CV of total mass concentration ratios was all less than 20%, with a mean value of 5.21% for all 20 particle size ranges (Fig. 5d). Thus, even with the presence of numerous confounding factors, our results indicate that aerosol uniformity in the test chamber was achieved in our AtoH test and errors associated with sampling position, sampling flow rate, filter extraction, and analysis were well controlled.

### Performance of test aerosol samplers

**Figure 6:**
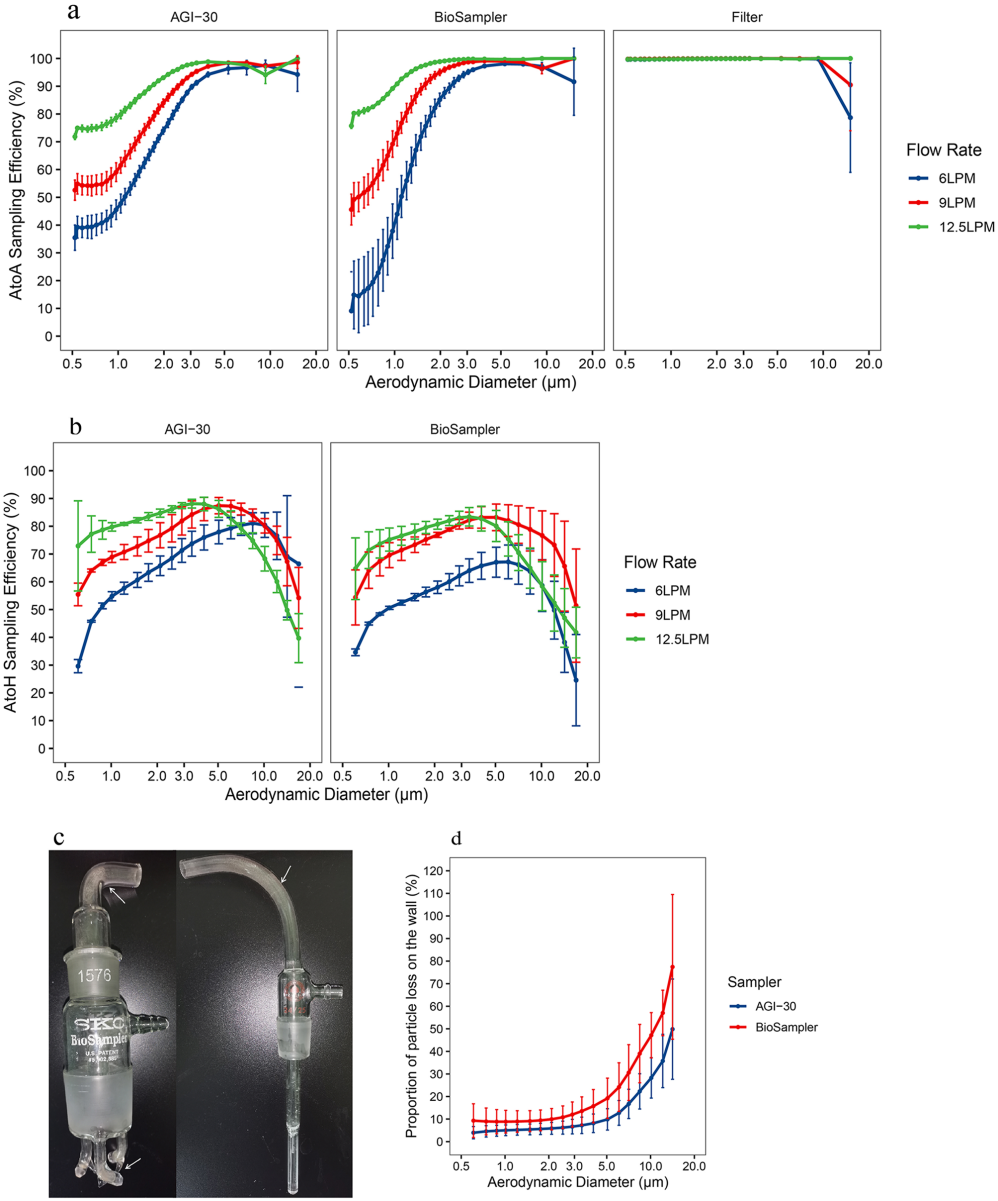
Performance of test aerosol samplers under different sampling flow rate. (**a**) AtoA sampling efficiency of three samplers operated at different flow rates as monitored by the APS. (**b**) AtoH sampling efficiency of two samplers operated at different flow rate as measured by the HELOS + CUVETTE system. (**c**) Deposition of particles on the inner surfaces of the sampler, with visible deposition indicated by white arrows. (**d**) Proportion of particle loss on the wall of AGI-30 and BioSampler as compared with polycarbonate filter under sampling flow rate 12.5 LPM. Log-scale was used for the x-axis of particle size distribution plots and error bars indicate SD of three replicate measurements.

In the present study, AtoA sampling efficiency of AGI-30 and BioSampler were tested at sampling flow rate of 6, 9 and 12.5 LPM (Fig. 6a). At 12.5 LPM, the AtoA sampling efficiency ranged from (74.95 ± 0.40) % at 0.54 μm to (100 ± 0.00) % at 10–20 μm for AGI-30 and (80.28 ± 0.53) % at 0.54 μm to (100 ± 0.00) % at 10–20 μm for BioSampler. Both the AGI-30 and BioSampler have great sampling efficiency at the recommended sampling flow rate of 12.5 LPM. However, there is a decrease in sampling efficiency for particles smaller than 3.9 μm with the decreasing of sampling flow rate. The AtoA sampling efficiency of AGI-30 for 0.54 μm particles dropped to (54.86 ± 3.66) % at 9 LPM and (39.24 ± 3.92) % at 6 LPM. But the AtoA sampling efficiency for 10–20 μm particles remained at (98.69 ± 2.31) % at 9 LPM and (94.24 ± 6.08) % at 6 LPM. The similar situation is observed with BioSampler. The AtoA sampling efficiency of BioSampler for 0.54 μm particles drops to (49.25 ± 6.01%) at 9 LPM and (14.84 ± 12.19) % at 6 LPM. The AtoA sampling efficiency for 10–20 μm particles remained at (100.00 ± 0.00) % at 9 LPM and (91.61 ± 12.07) % at 6 LPM. The results indicate that smaller particles are more difficult to be collected by impinger such as AGI-30 and BioSampler as compared to larger particles. And the influence of sampling flow seems to be greater in smaller particles. The 0.4 µm pore size polycarbonate filter showed superb AtoA sampling efficiency in all size range (0.5 to 20 μm). While the decreased AtoA sampling efficiency for all aerosol samplers in the 10–20 μm size range was ignored, because the scarcity of upstream particles and the re-aerosolization of particles deposited on the downstream pipeline may lead to the deviation of the AtoA sampling efficiency.

The results of the AtoH evaluation method present the different sampling efficiencies of AGI-30 and BioSampler, where lower sampling efficiency for large particles collected in the sampling liquid were observed for particle size above 4.0 μm (Fig. 6b). At a sampling flow rate of 12.5 LPM, the AtoH sampling efficiency increased from (72.91 ± 16.23) % at 0.6 μm to (88.00 ± 2.44) % at 4.0 μm and then decreased to (39.70 ± 8.83) % at 16.9 μm for AGI-30. This was also observed on the BioSampler, whose AtoH sampling efficiency increased from (64.71 ± 11.11) % at 0.6 μm to (83.18 ± 4.47) % at 4.0 μm and then decreased to (41.73 ± 9.14) % at 16.9 μm. As the sampling flow rate decreases, the AtoH sampling efficiency of AGI-30 for 0.6 μm particles were decreased to (55.45 ± 4.07) % at 9 LPM and (29.62 ± 2.38) % at 6 LPM. While the AtoH sampling efficiency for 4.0 μm particles remained at (86.21 ± 4.21) % at 9 LPM and (75.96 ± 4.53) % at 6 LPM. And the AtoH sampling efficiency for 16.9 μm particles increased to (54.20 ± 10.98) % at 9 LPM and (66.44 ± 44.38) % at 6 LPM. The BioSampler's sampling efficiency also varied with the sampling flow rate. The AtoH sampling efficiency of BioSampler for 0.6 μm particles were decreased to (54.36 ± 9.91) % at 9 LPM and (34.60 ± 1.22) % at 6 LPM. While the AtoH sampling efficiency for 4.0 μm particles remained at (83.18 ± 3.88) % at 9 LPM and (65.75 ± 4.83) % at 6 LPM. And the AtoH sampling efficiency for 16.9 μm particles increased to (51.42 ± 20.40) % at 9 LPM, but decreased to (24.57 ± 16.44) % at 6 LPM. At a sampling flow rate of 6 LPM, a swirling motion in the liquid collection medium cannot be created by BioSampler, which led to particles becoming deposited on the wall of the collection vessel rather than being drawn into the liquid collection medium. This could explain the low sampling efficiency of BioSampler for large particle when operated at 6 LPM. The results of AtoH sampling efficiency of impinger such as AGI-30 and BioSampler indicate that larger particles are more difficult to be collected into the sampling medium as compared to 4 μm particles. And the decreasing of sampling flow seemed to improve the performance of impinger’s AtoH sampling efficiency.

Reasons for the difference in the evaluation results of the AtoA and AtoH sampling efficiency could be the deposition of particles on the inner surfaces of the AGI-30 and the BioSampler, including the inside walls of the nozzles and the curves of the aerosol inlet, as observed (Fig. 6c). Wall-deposited particles in both samplers operated at 12.5 LPM were collected and quantified using laser diffraction analysis. Results showed that a large proportion of particles larger than 4 μm are present on the sampler inner wall, while particles smaller than 4 μm are rare (Fig. 6d). This indicated that particles larger than 4um tend to deposit on the inner surface of AGI-30 and BioSampler.

### Verification of the accuracy of the sampling efficiency evaluation method

To verify the accuracy of the evaluation method of AtoH sampling efficiency presented in this manuscript and also evaluate individual difference from other samplers, performance test using monodisperse aerosols and comparison of the results in this study with those of other literature were conducted. The results of monodisperse aerosols test presented high degree of consistency with the results evaluated by polydisperse aerosols (Table 1). P-values were all larger than 0.05 for all comparisons between the two kind aerosols. This result demonstrates the accuracy of the sampling efficiency evaluation when using polydisperse aerosols.

**Table 1:** Comparison of AtoH sampling efficiency evaluated by monodisperse and polydisperse aerosol.

Sampling Flow Rate (LPM)	Aerodynamic Diameter (μm)	AGI-30			BioSampler		
		FPSL (%)	ATD (%)	P value	FPSL (%)	ATD (%)	*P* value
12.5	0.77 or (0.74 ~ 0.88)	(75.56 ± 5.28)	(78.69 ± 3.36)	0.44	(73.26 ± 1.42)	(73.66 ± 5.10)	0.91
	1.1 or (1.01 ~ 1.21)	(81.25 ± 10.93)	(80.83 ± 0.49)	0.95	(82.91 ± 10.16)	(76.56 ± 3.41)	0.40
	1.9 or (1.75 ~ 2.09)	(87.44 ± 7.11)	(84.73 ± 1.45)	0.58	(88.14 ± 7.25)	(80.87 ± 1.81)	0.21
9	0.77 or (0.74 ~ 0.88)	(61.55 ± 7.63)	(66.98 ± 1.08)	0.34	(60.28 ± 8.42)	(67.46 ± 5.33)	0.29
	1.1 or (1.01 ~ 1.21)	(71.49 ± 6.78)	(70.72 ± 2.71)	0.87	(61.07 ± 8.42)	(71.41 ± 3.71)	0.16
	1.9 or (1.75 ~ 2.09)	(77.13 ± 5.79)	(76.77 ± 4.52)	0.94	(74.88 ± 5.52)	(76.89 ± 0.90)	0.59
6	0.77 or (0.74 ~ 0.88)	(43.97 ± 9.14)	(51.31 ± 1.03)	0.30	(36.31 ± 9.15)	(48.40 ± 0.37)	0.15
	1.1 or (1.01 ~ 1.21)	(56.06 ± 4.58)	(57.69 ± 2.16)	0.62	(49.87 ± 6.07)	(52.49 ± 0.86)	0.53
	1.9 or (1.75 ~ 2.09)	(64.95 ± 4.41)	(65.75 ± 3.52)	0.82	(60.51 ± 4.75)	(58.03 ± 2.23)	0.48

0.77 or (0.74 ~ 0.88) in the table were aerodynamic diameter of FPSL as statement and ATD particle as measured by laser diffraction method. Mean ± SD was used to indicate the range of sampling efficiency. Welch Two Sample t-test was used to assess the difference between evaluation results of monodisperse and polydisperse aerosol, and *p* < 0.05 was considered statistically significant.

**Figure 7:**
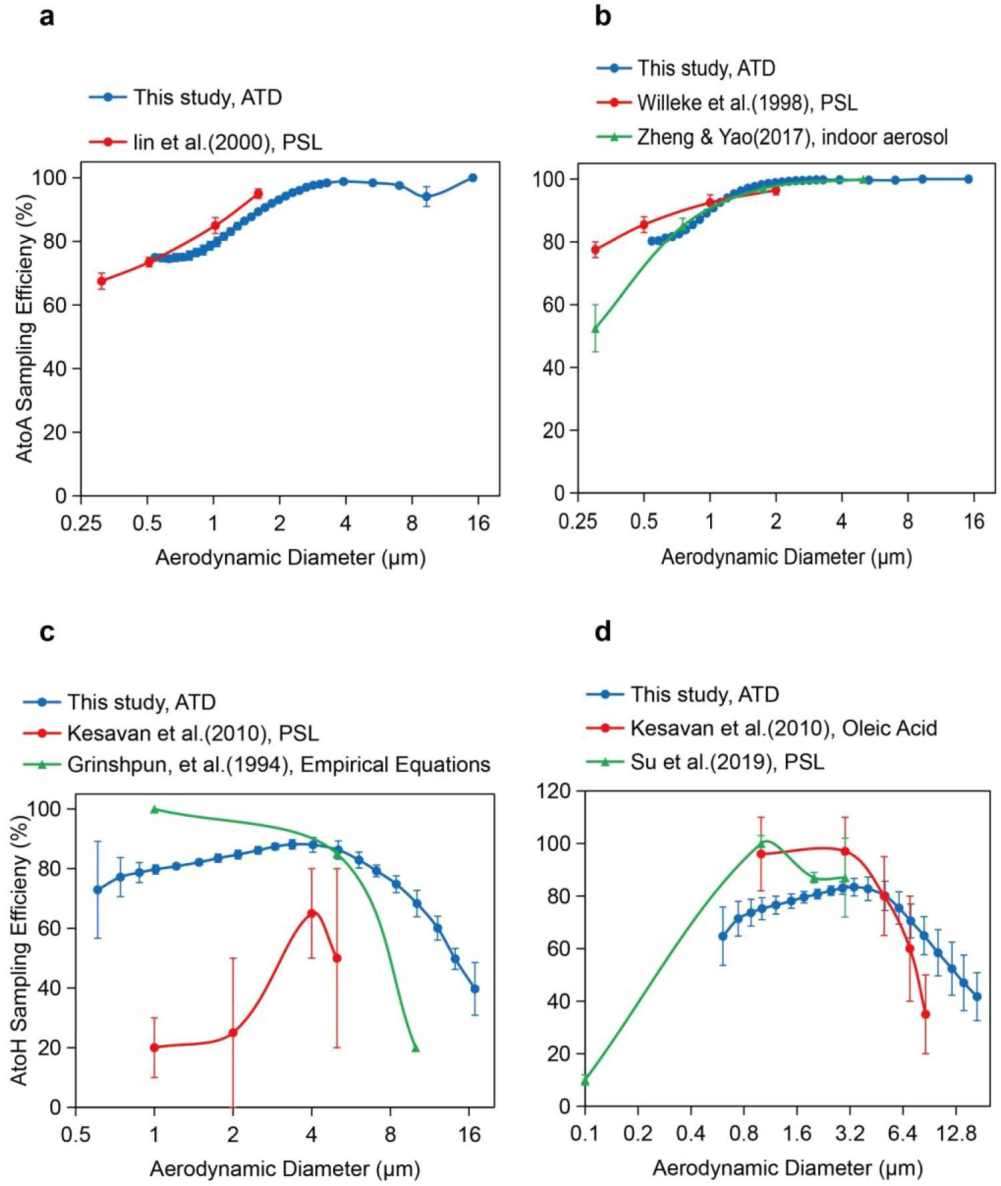
The size resolved physical sampling efficiency of AGI-30 and BioSampler retrieved from literature and results obtained in this work. Operating conditions were 12.5 L/min and 20 mL water. (**a**) AtoA Sampling efficiency of AGI-30. (**b**) AtoA Sampling efficiency of BioSampler. (**c**) AtoH Sampling efficiency of AGI-30 (**d**) AtoH Sampling efficiency of BioSampler.

Some theoretical and laboratory evaluation articles containing AGI-30 and BioSampler had been included for comparison^24,33,38–41^. Those studies were almost conducted in aerosol test chamber, aerosol test room or indoor environment, and monodisperse aerosols with only 3–5 sizes were used. In contrast, our evaluation method was able to test 20 particle sizes in the 0.6–16.8 µm range, which had a much wider and more continuous particle size range than other studies. The AtoA and AtoH sampling efficiency curves tested in this study showed a roughly similar trend to the curves tested in other reports for particle size range from 0.3 to 10 μm (Fig. 7). The AtoA sampling efficiency droped rapidly when particle was too small. While the AtoH sampling efficiency droped rapidly when particle was too large or too small, and the reason was claimed to be wall loss, rebound and re-aerosolization^41^. Overall, our results showed consistency with other studies, but there were still some variations. Differences in the use of aerosol particles, test methods, test equipment, detection methods and even sampler batches can contribute to different test results. The reported poor AtoH sampling efficiency of AGI-30 at particle size of 1–2 μm is questionable, because the AtoA sampling efficiency were around 80–90% and only a few wall loss were occurred at particle size of 1–2 μm for AGI-30.

## Discussion

Evaluation of aerosol sampler performance is a nontrivial task that must be performed with great care. However, the frequently used evaluation method is labor-intensive and time-consuming, owing to considerable number of experiments employing monodisperse aerosols spanning a range of sizes. This study attempts to address this limitation with the inexpensive, insoluble, easily dispersible polydisperse solid aerosols, which are capable of evaluating the size-resolved sampling efficiency in a single test. For this purpose, laser diffraction method is firstly developed for absolute mass quantification of ploydisperse solid particles in liquid collected from AtoH sampling test. Whereafter, a time-saving experimental methodology with sufficient accuracy is developed for evaluation of both AtoA and AtoH size-resolved sampling efficiency, which can be achieved respectively within 20 min and 90 min (laser diffraction analysis time was included in).

To determine the size distribution of particles in liquid, other studies have attempted to use the coulter counter to conduct post-sampling analysis in size-resolved AtoH evaluation, using polydisperse solid aerosols with an aerodynamic size range of 5–30 µm^31,42^. However, particle detection based on the coulter counter has inherent deficits in that the largest diameter particles in the test sample determine the smallest pore size of the aperture and limits the size range of detection ^43,44^. In the present study, the laser diffraction method is able to accurately measure the absolute mass concentration of test particles by introducing a reference sample to the laser particle analyzer detection process, the mean CV for accuracy ratios between fitted and measured result is within 4.9%, and the detection range is 0.61–118 µm of aerodynamic diameter (0.45–87.5 µm of equivalent diameter) which avoided the size range detection limitations of the coulter counter.

Evaluation of AtoA and AtoH sampling efficiency requires time stability and spatial uniformity of aerosol concentration respectively. To meet these requirements and achieve the goal of size-resolved evaluation of sampling efficiency for particle sizes ranging from 0.5 to 20 µm, ATD particles are used in this study. Compare with the previous studies used polydisperse liquid particles such as biological aerosols, which could change their shape and diameter during sampling and result in the bias of size resolved measurement, the insoluble solid polydisperse ATD particles are more suitable for evaluation of size-resolved sampling efficiency because of their stable physical characteristics^45–47^. Since the concentration of generated solid aerosols is not as stable as that of generated liquid aerosols, our experimental set-up is intentionally designed as a good strategy for minimizing the impact of this challenge. From the results of validation, the mean ratio of total count concentration between upstream and downstream sampling periods in AtoA sampling test is approximately 100%, and the mean ratio of mass concentration between two sampling locations in AtoH sampling test is 97.5%. These results prove that aerosol stability and uniformity have been achieved in our experimental methodology. In addition, a phenomenon of large particle deposition in the test chamber was observed, that may be the reason for the relatively large variation in aerosol concentrations for particles above 10 µm, and also leads to a narrow aerodynamic particle size range of 0.6–16.9 µm for samples collected in the AtoH sampling test while ATD aerosols with aerodynamic particle size of 0.6—27 µm are generated. In this case, an aerosol wind tunnel would be more suitable for sampling performance tests of large particles, because of its stability and uniformity for a wide diameter range of aerosols in testing^31,48,49^. Nevertheless, the detection size range of both the AtoA and AtoH sampling tests in this study are wide enough for testing the respirable and thoracic samplers ^50,51^.

The evaluation of AtoA and AtoH sampling efficiency reveal the performance characteristics of the samplers for different conditions. Evaluation of AtoA sampling efficiency confirms that most particles in our test size range can be collected by the AGI-30 and the BioSampler at a sampling flow rate of 12.5 LPM, as others reported^24,33,38^. However, evaluation of AtoH sampling efficiency indicated that large particles collected by the sampler are more likely to deposit on the inner surface of the sampler rather than in the sampling medium, as others reported^39–41^. A comprehensive understanding of AtoA and AtoH sampling efficiency for aerosol samplers can provide essential information when conducting aerosol sampling in field. In the present study, evaluations of AtoH sampling efficiency show less repeatability than evaluations of AtoA sampling efficiency because of the elaborate experimental steps that cause errors associated with particle sampling, transportation, handling and analysis.

As the key influencing factor of sampling aerosols, sampling flow rate has a significant effect on the total volume of sampling aerosol, sampling efficiency and biological sampling stress^52,53^. In our study, improvement of the total particle sampling efficiency of the AGI-30 and the BioSampler presented by the evaluation of AtoA sampling efficiency is achieved by increasing the sampling flow rate, but at the cost of increasing deposition on the inner surface of aerosol sampler as described in evaluation of AtoH sampling efficiency. To fully understand the performance of test aerosol sampler, it is necessary to compare the performance of the sampler under different conditions.

In conclusion, our experiments demonstrate that evaluation of size-resolved sampling efficiency of aerosol sampler can be measured by the laser diffraction method with sufficient accuracy. The advantages of shorter time required and wide evaluation range of aerodynamic particle sizes at a relatively low cost make the AtoA and AtoH experimental procedure and laser diffraction method particularly suitable for sampler evaluation. In addition, the combined evaluation of AtoA and AtoH sampling efficiency provides more detailed performance indicators for the samplers.

## Supplementary Information

Supplementary Information.
