# Supplementary material for: An experimental method for efficiently evaluating the size-resolved sampling efficiency of liquid-absorption aerosol samplers
Source: Sci Rep. 2022 Mar 18;12:4745. doi: 10.1038/s41598-022-08718-8 (PMC8932469; doi:10.1038/s41598-022-08718-8)
Supplement: Supplementary file 1 — Supplementary Information. [file 41598_2022_8718_MOESM1_ESM.doc]

**Supplementary Information**

**An experimental method for efficiently evaluating the size-resolved sampling efficiency of liquid-absorption aerosol samplers**

Jianshu Guo ^1†^, Xinying Zheng^1†^, Tongtong Qin^1,2^, Meng Lv^1^, Wei Zhang^1^, Xiaolin Song^1^, Hongying Qiu^1^, Lingfei Hu^1^, Lili Zhang^1^, Dongsheng Zhou^1^, Yansong Sun^1*^, Wenhui Yang^1*^

^1^State Key Laboratory of Pathogen and Biosecurity, Beijing Institute of Microbiology and Epidemiology, Beijing, China

^2^Laboratory Animal Center, Academy of Military Medical Science, Beijing, China

^†^ These authors contributed equally to this work.

*Correspondence should be addressed to:fionyoung@163.com; [sunys1964@hotmail.com](mailto:sunys1964@hotmail.com)

Number of pages: 2

Number of tables: 2

**Table S1 Characteristics of recovery efficiency of ATD particles extraction from polycarbonate filter**

| Repeat | Pre-sampling (mg) | Post-sampling (mg) | Post-extraction (mg) | Recovery  Efficiency  (%) |
| --- | --- | --- | --- | --- |
| 1 | 11.08 | 32.37 | 11.12 | 99.81% |
| 2 | 10.82 | 27.56 | 10.88 | 99.64% |
| 3 | 10.91 | 29.88 | 10.93 | 99.89% |

Polycarbonate filter was used to sampling ATD particles in aerosol test chamber for 10min. And weighing of dried filter membranes of pre-sampling, post-sampling and post-extraction were performed using semi-micro balances(sartorius).

**Table S2 Characteristics of recovery efficiency of FPSL particles extraction from polycarbonate filter**

| Particle Size  (μm) | Dilution  Solution | Extracted Solution | | | Recovery  Efficiency  （%） |
| --- | --- | --- | --- | --- | --- |
|  |  | Rep1 | Rep2 | Rep3 |  |
| 0.77 | 2297.29 | 2158.78 | 2289.07 | 2305.44 | (97.99±3.50) |
| 1.1 | 1936.80 | 1800.52 | 1999.38 | 1940.11 | (98.79±5.27) |
| 1.9 | 1832.57 | 1922.57 | 1758.82 | 1952.01 | (102.47±5.68) |

20μl FPSL was added on the surface of polycarbonate filter membrane and dried at 50 ℃. Then FPSL particles attached to polycarbonate filter membrane were extracted and suspended in 50ml of extracted solution. Fluorescence intensity of dilution and extracted of FPSL were measured by Qubit 4 with excitation wavelength at 470nm.
